# Epithelial competition determines gene therapy potential to suppress Fanconi Anemia oral cancer risk

**DOI:** 10.1101/2025.02.26.640284

**Published:** 2025-02-27

**Authors:** Hunter L. Colegrove, Raymond J. Monnat, Alison F. Feder

**Affiliations:** 1 Department of Genome Sciences, University of Washington, Seattle, WA; 2 Department of Laboratory Medicine and Pathology, University of Washington, Seattle, WA; 3 Department of Bioengineering, University of Washington, Seattle, WA; 4 Herbold Computational Biology Program, Fred Hutch Cancer Center, Seattle, WA

## Abstract

Fanconi Anemia (FA) is a heritable syndrome characterized by DNA damage repair deficits, frequent malformations and a significantly elevated risk of bone marrow failure, leukemia, and mucosal head and neck squamous cell carcinomas (HNSCC). Hematopoietic stem cell gene therapy can prevent marrow failure and lower leukemia risk, but mucosal gene therapy to lower HNSCC risk remains untested. Major knowledge gaps include an incomplete understanding of how rapidly gene-corrected cellular lineages could spread through the oral epithelium, and which delivery parameters are critical for ensuring efficient gene correction. To answer these questions, we extended an agent-based model of the oral epithelium to include the delivery of gene correction *in situ* to FA cells and the competitive dynamics between cellular lineages with and without gene correction. We found that only gene-corrected lineages with substantial proliferative advantages (probability of resisting displacement out of the basal layer ≥ 0.1) could spread on clinically relevant timelines, and that these lineages were initially at high risk of loss in the generations following correction. Delivering gene correction to many cells minimizes the risk of loss, while delivery to many distinct locations within a tissue maximizes the rate of spread. To determine the impact of mucosal gene therapy in preventing the clonal expansion of pre-cancerous mutations, we compared the expected burden of TP53 mutations in simulated tissue sections with and without gene correction. We found that when FA cells have elevated genome instability or a TP53-dependent proliferative advantage, gene correction can substantially reduce the accumulation of pro-tumorigenic mutations. This model illustrates the power of computational frameworks to identify critical determinants of therapeutic success to enable experimental optimization and support novel and effective gene therapy applications.

## Author summary

We investigated factors influencing the success of oral mucosal gene therapy for Fanconi Anemia (FA), a genetic syndrome marked by DNA repair defects in conjunction with a heightened risk of cancer. We used a computational model of the oral epithelium to determine how gene therapy corrected cells compete with FA background cells and the best gene delivery approaches to promote effective tissue replacement by gene-corrected cells. We find that gene-corrected cells require strong proliferative advantages to spread effectively, and that initially delivering gene correction to more cells reduces the chance that these cells are stochastically eliminated before they can spread. We also demonstrate that gene correction reduces the accumulation of pro-tumorigenic TP53 mutations in an FA context, where genomic instability can elevate the mutation rate and FA-specific selective pressures could favor accelerated TP53 clonal expansion. This research provides a useful framework for guiding mucosal gene therapy experiments and the development of effective oral gene therapy protocols for cancer prevention in FA.

## Introduction

Fanconi Anemia (FA) is an inherited genetic disorder and cancer predisposition syndrome linked to increased risk of bone marrow failure, leukemia, and epithelial cancers. FA arises from biallelic pathogenic mutations in any of 23 FANC genes that work in concert as a pathway (the ‘FA pathway’) to detect and coordinate the repair of many types of DNA damage including DNA interstrand crosslinks and double-strand breaks (1–3). This compromised repair capacity is associated with an elevated risk of certain cancers, particularly head and neck squamous cell carcinomas (HNSCC). FA patients experience HNSCC rates several hundred to over a thousand fold greater than the general population (4–8), and a corresponding elevation in mucosal anogenital squamous carcinomas. These cancers often contain pathogenic mutations in TP53, a crucial tumor suppressor gene (9–11), and are genomically unstable. Further, they are difficult to treat via standard care chemotherapy and radiation due to the FA-associated constitutional DNA repair defect (8,12–14).

**Figure 1.**
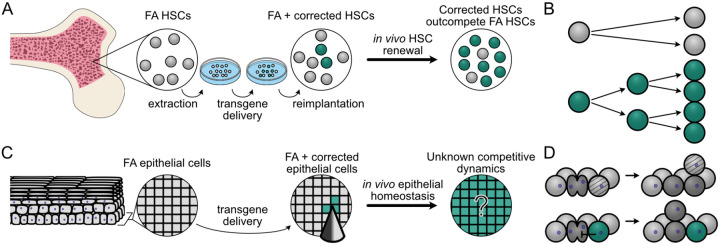
Modeling gene therapy in FA HSCs and oral epithelium **(A)** Hematopoietic stem cell (HSC) gene therapy in FA. FA HSCs (grey) are harvested from FA patient bone marrow, corrected *ex vivo* (green), then re-implanted to expand *in vivo*. **(B)** Corrected HSCs (green) have a proliferative advantage compared to FA HSCs (grey) and outcompete uncorrected HSCs over time *in vivo*. **(C)** Oral mucosal gene therapy in FA. *In situ* gene delivery (here via microneedles) into oral epithelium to correct the underlying FANC gene defect. Over time, corrected cells (green) may or may not clonally expand within FA oral epithelium to replace FA host cells (grey). **(D)** In our model, FA epithelial cells (light grey) are displaced (light grey with bands) by dividing FA cells (dark grey, top), in contrast to gene-corrected epithelial cells (green) that can resist displacement by dividing neighbors (dark grey) to persist and expand in the basal layer proliferative niche (bottom).

Given these challenges, corrective gene therapy represents a promising strategy for the prevention of FA-associated HNSCC. This strategy has already shown success in FA-associated bone marrow failure, where gene-corrected hematopoietic stem cells (HSCs) can reverse marrow failure and suppress the risk of developing leukemia: HSCs are removed, the specific mutant FANC gene is corrected *ex vivo*, and corrected cells are re-implanted into the bone marrow (Figure 1A). Recent FA gene therapy clinical studies have found that gene-corrected FA HSCs possess a proliferative advantage over uncorrected FA HSCs (Figure 1B, (15–19)). As a result, small numbers of implanted corrected HSCs have been shown to expand over time to reverse FA-associated bone marrow failure.

The success of HSC gene correction in treating marrow failure suggests that oral gene correction may provide a parallel way to prevent FA-associated HNSCC. However, gene correcting mucosal epithelial cells may prove more challenging than HSCs due to the constraints of tissue architecture. Epithelial tissues are tightly organized, which could limit corrected cell expansion when compared with HSCs in bone marrow (Figure 1C). However, cellular lineages can clearly expand within epithelial tissues, e.g., those lineages harboring cancer driver mutations (20–22), or spontaneous revertant mutations in the context of heritable skin diseases such as epidermolysis bullosa (23–25). Thus understanding the degree of proliferative advantage required for FA-corrected oral epithelial cells to expand and persist will be essential in devising mucosal gene correction protocols for FA epithelium.

A second major challenge of preventative gene therapy against FA-associated HNSCCs is that oral epithelial cells must be corrected *in situ* rather than removed and reimplanted (Figure 1C). This will require identifying efficient mucosal gene correction protocols that favor the expansion and persistence of gene-corrected epithelial cells. One promising new approach is to adapt microneedle arrays originally developed for mucosal vaccination to deliver gene correction reagents (26–31). Microneedle arrays can deliver customizable doses of correction reagents in different spatial configurations, but the optimal array configurations and microneedle properties required to achieve efficient delivery will need to be determined.

Computational modeling provides an efficient framework in which to integrate both different delivery approaches and the effect of epithelial structure on gene correction to optimize therapeutic success. Agent-based models have a long history of providing valuable insight into cell-cell competition and the emergent spatial dynamics shaping tissue behavior (32–34). We therefore extended an existing agent-based model of squamous epithelium (35) to include gene correction delivered by arrays of diffusible microneedles in order to answer several key questions: 1) what degree of proliferative advantage must corrected cells possess to persist and spread effectively in the oral epithelium; 2) how do different gene correction efficiencies and spatially-structured delivery strategies such as microneedle arrays affect long-term gene correction success; and 3) can mucosal gene correction prevent the clonal spread of pathogenic TP53 mutations and the progression to oral cancer in individuals with FA? Our results provide a framework for understanding the dynamics of gene therapy in FA oral epithelium, and how best to use mucosal gene correction to reduce cancer risk and improve FA patient health outcomes.

## Results

### *In silico* model of gene correction in the oral epithelia

In order to simulate the impact of gene therapy in epithelial tissues we employed the HomeostaticEpidermis model, a three-dimensional lattice-based hybrid cellular automaton originally developed to model epidermal dynamics over human lifespans (35). In this model, each epithelial cell is an independent agent whose rate of division and death are determined by a diffusible growth factor emanating from the basal layer to simulate a fibroblast source. Dividing cells reside on a basal layer where the resultant progeny are displaced upward, undergo terminal differentiation and are eventually shed at the epithelial surface. This model robustly captures key epithelial features including structure, homeostasis with cell turnover, and realistic clonal expansion of cells that resist death or differentiation.

We adapted the HomeostaticEpidermis model to explore FA oral gene therapy by introducing simulated gene correction that confers a potential proliferative advantage over uncorrected FA cells. In stratified squamous epithelium, clonal expansion can occur via progenitor cell fate imbalance in the basal layer (36–38). This proliferative advantage was modeled via a persistence coefficient, $p_{\text{corr}}$, which represents the probability that a corrected cell resists displacement out of the basal layer during neighbor cell divisions (Figure 1D). By preventing displacement, corrected cells remain in the basal layer progenitor niche and retain their proliferative capacity. This mirrors the approach from Schenck et al. for modeling the proliferative advantage of *NOTCH1* mutations in skin (35), and permitted us to explore the potential effect of gene correction in an FA background where gene-corrected cells had anywhere from no advantage (or a neutral $p_{\text{corr}}=0$) to an extremely beneficial persistence coefficient ($p_{\text{corr}}=1$). We first investigated the proliferative advantage necessary to promote reliable expansion of corrected cellular lineages in an FA mucosal background.

### Gene correction requires a strong proliferative advantage to spread over clinically relevant time periods

**Figure 2.**
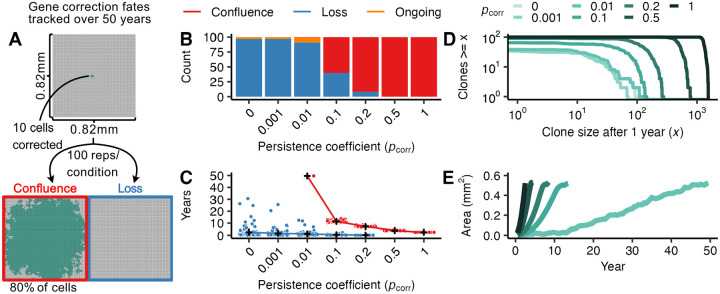
Mucosal gene therapy depends on a strong proliferative advantage of gene-corrected cells. **(A)** 10 gene-corrected cells (green) were tracked over 50 years in a 0.67 mm^2^ FA mucosal tissue section (100 replicate simulations per condition). The gene correction fates were tracked to identify simulations reaching confluence (80% of basal layer cells corrected, red box) or loss (no remaining gene-corrected cells, blue box). **(B)** Number of simulations achieving confluence (red), loss (blue), or ongoing expansion (orange) at 50 years as a function of persistence coefficient $\left(p_{\text{corr}}=0,0.001,0.01,0.2,0.5,1\right)$. **(C)** Time at which outcomes in (B) reached confluence or loss as a function of persistence coefficient, with mean times for each $p_{\text{corr}}$ indicated in black (+) and outcome of individual simulations as points. Gene-corrected patches not reaching confluence or loss by 50 years are not plotted. (D) Number and size of gene-corrected patches at 1 year as a function of persistence coefficient $p_{\text{corr}}$. (E) Average area of confluent gene-corrected patches as a function of time and persistence coefficient $p_{\text{corr}}$.

We hypothesized that gene-corrected cells will require a proliferative advantage compared to uncorrected FA cells to spread through FA oral mucosa. To set baseline expectations for cellular competition without a proliferative advantage, we first considered the case in which a corrected cell has equivalent division behavior to the FA background: that is, when $p_{\text{corr}}=0$. We simulated 0.67 mm^2^ (100 × 100 cells) FA tissue sections and delivered gene correction to 10 cells clustered in the tissue center (mirroring a single microneedle delivery, Figure 2A). Because the median age of developing solid tumors for people with FA is ~30 years (5,7), we reasoned that corrected cells would need to displace FA cells in the first decade or two after gene correction to be most clinically impactful. We thus followed simulated tissue sections for up to 50 years to determine the fate of corrected cells and their descendants (Figure 2A). We considered three fates: all corrected cells could be displaced from the basal layer (i.e., “loss”); corrected cells could overtake 80% or more of the basal layer (i.e., “confluence”); or neither of these outcomes might be reached (i.e., “ongoing” dynamics). Under neutral conditions ($p_{\text{corr}}=0$), nearly all simulations (97%) led to the loss of corrected cells by 50 years (Figure 2B) with most corrected cell loss occurring within months of correction (Figure 2C). In the remaining 3% of simulations, neutral corrected patches remained modestly sized; the largest corrected clone reached only 0.15 mm^2^. This result indicates that in the absence of a proliferative advantage, gene correction will not efficiently convert FA epithelium.

We next investigated how increasing proliferative advantages might enable corrected cells to spread more efficiently through epithelium on clinically-relevant timelines. Because we had little prior knowledge about the degree of proliferative advantage conferred by FANC gene correction, we investigated a wide range of persistence coefficients ranging from unlikely to guaranteed persistence ($p_{\text{corr}}=0.001,0.01,0.1,0.2,0.5,1$). In all experiments, FA cells did not resist displacement (i.e., had a persistence coefficient of 0).

As expected, larger persistence coefficients decreased the probability of corrected cell loss (Figure 2B), where persistence coefficients of $p_{\text{corr}}\geq 0.1$ were required to avoid most loss events. When loss did occur at large persistence coefficients, it occurred early, with 88% of those losses within the first year after gene correction (Figure 2C). This is analogous to the establishment frequency in population genetics, where once beneficial mutations reach a certain frequency, loss becomes increasingly unlikely. Gene correction with persistence coefficients closer to neutrality can persist for years or decades before loss, but will rarely expand to any significant degree. Full clone size distributions one year after correction are shown in Figure 2D.

For gene-corrected cells that escape loss, larger persistence coefficients also increased the speed of corrected cell spread. When corrected cells with smaller persistence coefficients ($p_{\text{corr}}<0.1$) escaped loss, they almost never reached confluence in the tissue sections by 50 years. In contrast, gene correction with $p_{\text{corr}}\geq 0.1$ that avoided early loss always reached confluence in the tissue sections by 50 years (Figure 2B). The time at which populations reached confluence decreased as the persistence coefficient increased (Figure 2C), with $p_{\text{corr}}=1$ growing at approximately five times the speed of $p_{\text{corr}}=0.1$ (0.25 ± 0.027 *SE* mm^2^/year versus 0.048 ± 2.0 ⋅ 10^−3^ *SE* mm^2^/year, Figure 2E). In contrast, rates of expansion of corrected patches with very small persistence coefficients ($p_{\text{corr}}\leq 0.01$) would require more than 80 years to reach 1 mm^2^. These results collectively indicate that to be effective, oral mucosal gene correction must confer a substantial proliferative advantage ($p_{\text{corr}}\geq 0.1$) to persist and spread on clinically-relevant timescales.

### Optimizing transgene delivery for maximal corrected tissue coverage

Having demonstrated above that individual gene-corrected patches with a strong proliferative advantage could spread locally, we next considered how patch spread was determined by transgene dose and the spatial configuration of the cells corrected. We investigated the case in which transgene delivery by resorbable microneedles allows both gene dose and in-tissue diffusibility to be modified as part of microneedle design and fabrication.

**Figure 3:**
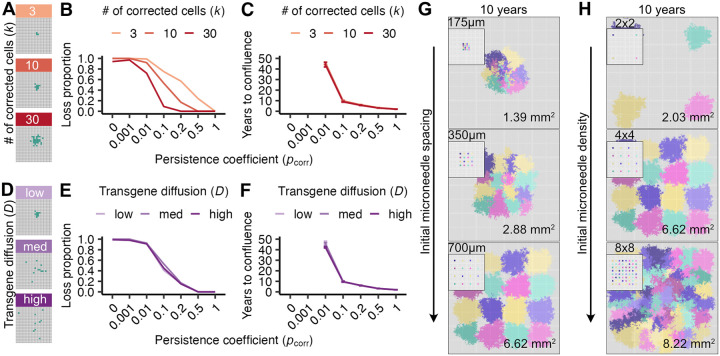
Higher corrected cell numbers and optimal spacing can increase the likelihood of tissue replacement despite clonal interference. **(A)** Initial spatial distribution of gene-corrected cells (k=3,10,30 cells, in green) with a constant diffusion coefficient (D=2) in tissue sections of the same area. **(B)** Probability of gene-corrected patch loss as a function of persistence coefficient ($p_{\text{corr}}$) and initial corrected cell number (k). **(C)** Time to confluence (>80% takeover of the basal layer) of gene-corrected patches as a function of persistence coefficient ($p_{\text{corr}}$) and initial corrected cell number (k). **(D)** Initial spatial distribution of ten gene-corrected cells (green) as a function of diffusion coefficient (D=2,10,20, corresponding to low, medium and high diffusion) across tissue sections of the same area. **(E,F)** correspond to **(B,C)** but contain ten initially corrected cells with varying transgene diffusion (D) values. For **(B,C,E,F)**, 100 simulations per condition were run for each persistence coefficient $p_{\text{corr}}$, with error bars indicating interquartile ranges. Visualizations of gene-corrected cell patches ten years after correction as a function of microneedle spacing **(G)** or density **(H)** on 10.67 mm^2^ tissue sections. Initial delivery array configurations are shown in panel insets, and each color represents the descendants of a single microneedle that corrects k=30 cells with D=2. Areas of corrected cell patches at ten years are quantified in the lower right corner of each tissue section. In **(G)**, 4×4 microneedle arrays were used with interneedle spacings of 175 μm, 350 μm, and 700 μm. In **(H)**, 2×2, 4×4, and 8×8 microneedle arrays were used with a constant delivery area of 4.43 mm^2^, corresponding to microneedle densities of 0.90, 3.61, and 14.44 microneedles/mm^2^, respectively.

We first investigated how a greater number of corrected cells, equated here to transgene dose, affected the probability of avoiding corrected cell loss and the time to confluence when delivered in a single compact patch (Figure 3A). As expected, we found that correcting more cells decreased the probability of loss across persistence coefficients (Figure 3B), where 30 as opposed to 10 corrected cells decreased the probability of loss for $p_{\text{corr}}=0.1$ from 50% to 10%. However, increasing the number of corrected cells did not affect time to confluence when conditions favored reaching confluence (Figure 3C). This finding reflects that the time needed to grow from 3 cells to 30 is short compared to the time needed to overtake the majority of cells in a tissue (i.e., 10 years for $p_{\text{corr}}=0.1$). Therefore averting early corrected cell loss is the primary advantage of larger doses within a single injection site.

We next investigated how transgene diffusion from its point of injection might improve corrected cell spread by initially correcting cells over a greater tissue area. This effect might allow corrected cells to more effectively compete for space against FA cells as opposed to other gene-corrected ones. We modeled transgene dispersal away from a point of delivery with an approximation to Brownian diffusion with diffusion constant D (see Materials and Methods), and examined D∈(2,10,20) to capture low, intermediate and high degrees of spatial diffusion when starting from 10 corrected cells (Figure 3D). Surprisingly, we found that increased diffusibility did not affect the probability of loss of a corrected patch or its rate of spread (Figure 3EF). This finding may reflect that early stochastic loss of individual corrected cells is more likely driven by being displaced by neighboring cells (regardless of correction status), rather than their failure to displace corrected neighboring cells at division. Thus, transgene delivery to enable wider transgene diffusion from a single microneedle source is unlikely to substantially improve the spread of corrected cell patches.

A key advantage of microneedles is their ability to be arrayed to optimize transgene delivery spacing and density. In light of finding that transgene diffusibility minimally affected the rate of spread from single needles, we investigated how the patterning of microneedle arrays could be used to optimize transgene delivery and tissue correction over a 10 year period.

We first asked how needle spacing might promote - or avoid - competition between corrected cells and their resultant rate of spread within a tissue section. We reasoned that each individual microneedle could be engineered to have a low correction loss probability, and therefore interference between microneedle sites could be more important than the interference between cells as illustrated in Figures 3D-F. We thus examined transgene delivery, corrected cell persistence and rate of spread using a 4×4 configuration of 16 low diffusibility (D=2) microneedles in which each needle corrected 30 cells to confer a strong proliferative advantage ($p_{\text{corr}}=0.1$). We explored needle spacings of 175 μm, 350 μm, and 700 μm to reflect commonly engineered interneedle distances (26–31) with the aim of gene-correcting a 10.67 mm^2^ tissue section (Figure 3G). We found that the most widely spaced microneedle arrays corrected more than five times as much tissue area in a ten year period than the most tightly spaced arrays for a given number of microneedles. In tightly spaced arrays, corrected cells at the perimeter expanded more than those in the interior; this apparent competition among corrected cells was not observed in the most widely spaced arrays. Thus appropriate transgene delivery spacing has the potential to minimize interference or competition of corrected cells and maximize the area of corrected tissue.

We also investigated whether higher microneedle array densities than those explored above could influence the likelihood of confluence within a given delivery area. This is a practically important question as arrays can be engineered with substantially higher densities than the ones we examined above. In our previous experiment, tightly spaced arrays led to a slower rate of confluence but achieved greater localized tissue coverage than did widely spaced arrays (Figure 3G). We found that increasing needle densities led to more complete conversion of a given tissue area and did not limit the approach to confluence (Figure 3H). For example, increasing microneedle density to 8×8 on a fixed backing size of 4.43 mm^2^ increased tissue correction by ~25% whereas decreasing microneedle density to 2×2 on the same backing patch size decreased the tissue correction by ~70%. These results collectively indicate that dense microneedle array transgene delivery in conjunction with a strong proliferative advantage can provide widespread tissue level correction even when promoting potential spatial competition among corrected cells and clones.

### Gene correction reduces ${TP53}^{-}$ clonal expansion in genomically unstable FA epithelium

**Figure 4.**
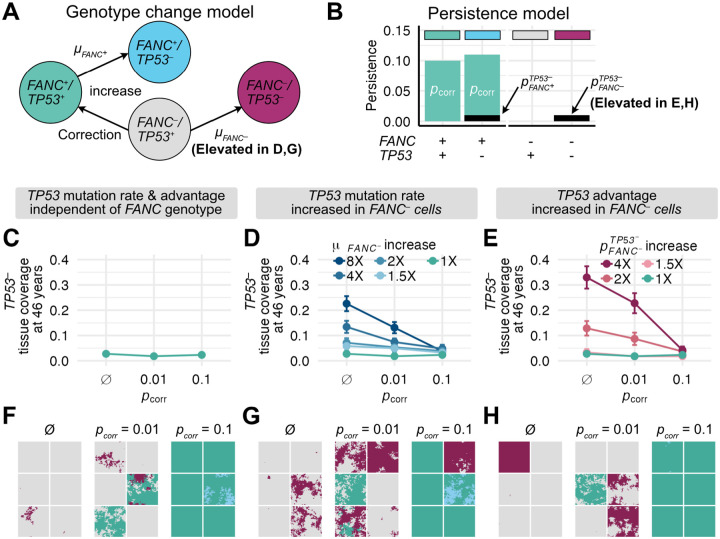
Gene correction reduces spread of TP53 mutations through FA tissue. **(A)** Genotype change model of four cellular genotypes at two loci: background FA cells ($FANC^{-}{/TP53}^{+}$) in grey; FA cells with TP53 mutations ($FANC^{-}{/TP53}^{-}$) in magenta; gene-corrected cells ($FANC^{+}{/TP53}^{+}$) in green; gene-corrected cells with TP53 mutations ($FANC^{+}{/TP53}^{-}$) in cyan. Arrows indicate genotype transitions that can occur via either mutation or gene correction. **(B)** Genotype-specific persistence coefficients from **(A)**. **(C-H)** ${TP53}^{-}$ tissue coverage at 46 years in 0.33 mm^2^ simulated tissue sections as a function of gene correction, comparing no correction (∅) to correction with $p_{\text{corr}}=0.01$ or $p_{\text{corr}}=0.1$. 100 simulations were performed for each condition at a given persistence coefficient except in panels **C,F** where 300 simulations were performed. Panels **C-E** show the proportion of tissue coverage at 46 years with **(C)** comparable TP53 mutation rates and persistence coefficients regardless of FA genotype, **(D)** an elevated TP53 mutation rate in $FANC^{-}$ cells ($\mu_{FANC^{-}}=m\cdot \mu_{FANC^{+}},m=(1.5,2,4,8)$) with equivalent TP53 persistence coefficients regardless of FANC genotype or **(E)** equal TP53 mutation rates in $FANC^{-}$ and $FANC^{+}$ cells, with elevated TP53 persistence coefficients in $FANC^{-}$ cells $\left(p_{FANC^{-}}^{TP53^{-}}=r\cdot p_{FANC^{+}}^{TP53^{-}}r=(1.5,2,4)\right.$). Error bars represent standard errors. Panels **F-H** represent six representative tissue sections at 46 years from **C-E**, with colors corresponding to genotypes described in (**A**): (**G**) represents a simulated 8-fold increase in $\mu_{FANC^{-}}$, and **(H)** a 4-fold increase in $p_{FANC^{-}}^{TP53^{-}}$.

Above, we investigated conditions under which FA gene-corrected cells can spread through the FA oral epithelium. How might the spread of corrected cells counter HNSCC risk or progression? An early stage in the development of FA-associated and sporadic HNSCCs is the clonal expansion of pathogenic mucosal TP53 mutations (9–11). We thus asked if FA gene correction could counter the spread of TP53 mutations in oral epithelium. In our model, mutations in a single copy of TP53 can occur in either corrected (here denoted $FANC^{+}$) or FA (here denoted $FANC^{-}$) cells according to mutation rates $\mu_{FANC^{+}}$ and $\mu_{FANC^{-}}$ (Figure 4A), and these TP53 mutations confer persistence coefficient advantages of $p_{FANC^{+}}^{TP53^{-}}$ and $p_{FANC^{-}}^{TP53^{-}}$, respectively. As before, corrected $FANC^{+}$ cells without TP53 mutations have a persistence coefficient of $p_{\text{corr}}$, so corrected $FANC^{+}$ cells with a TP53 mutation have a total persistence coefficient of $p_{\text{corr}}+p_{FANC^{+}}^{TP53^{-}}$ (Figure 4B). We estimated a mutation rate ($\mu_{FANC^{+}}$) and persistence coefficient ($p_{FANC^{+}}^{TP53^{-}}$) of TP53 mutations in a $FANC^{+}$ background based on the rate of occurrence and size of TP53 clones in healthy human esophageal tissue (see Materials and Methods). Because few data are available on the rates of TP53 mutation and spread in $FANC^{-}$ tissues, we initially assumed that the rates of occurrence and clonal expansion of TP53 mutations were similar in $FANC^{+}$ and $FANC^{-}$ tissues (i.e., $\mu_{FANC^{+}}=\mu_{FANC^{-}}$ and $p_{FANC^{+}}^{TP53^{-}}=p_{FANC^{-}}^{TP53^{-}}$). We reasoned that even if corrected and uncorrected cells produced the same number of TP53 mutations with comparable proliferative advantages, gene correction might provide a benefit by making TP53 mutant cells less able to displace $FANC^{+}$ as opposed to $FANC^{-}$ cells, thus restricting their expansion potential.

To test this idea, we asked if gene correction could limit TP53 mutational prevalence in FA tissue when TP53 mutation rates and proliferative advantages were comparable for $FANC^{-}$ and $FANC^{+}$ cells (Figure 4CF). We simulated 0.33 mm^2^ (70×70 cells) tissue sections in which a single microneedle gene corrected 30 cells (D=2) with persistence coefficients of $p_{\text{corr}}=0.01$ or $p_{\text{corr}}=0.1$ at the tissue center, then followed these sections up to 50 years. We compared these corrected tissue sections to uncorrected tissue section controls. Surprisingly, we found that TP53 mutations reached similar tissue abundances over 46 years regardless of gene correction status (Figure 4CF). While gene correction restricted ${TP53}^{-}$ spread in $FANC^{-}$ cells, mutations still arose and expanded within corrected $FANC^{+}$ cells, leading to similar overall tissue abundances (compare cyan $FANC^{+}/{TP53}^{-}$ cells with $p_{\text{corr}}=0.1$ in Figure 4F tissue sections to uncorrected magenta $FANC^{-}/{TP53}^{-}$ cells). Gene correction might nonetheless be beneficial if $FANC^{-}/{TP53}^{-}$ double mutants are more likely to progress to cancer than $FANC^{+}/TP53^{-}$ mutants, but it is unlikely to lower the overall ${TP53}^{-}$ burden if TP53 mutation rates and proliferative advantages are comparable between corrected and uncorrected tissue. Full 46-year tissue coverage trajectories from these simulations are shown in Supplemental Figure 1A-C.

The above results assume that the TP53 mutation rate and mutant proliferative advantage are identical regardless of FANC genotype. However, the dramatically increased risk of HNSCC in FA individuals relative to non-FA individuals could reflect genomic instability that creates more pathogenic mutations, or the more rapid spread of pathogenic mutations through FA epithelia, or both. We therefore investigated how FA functional status could affect TP53 mutation production or spread to alter the outcome of gene correction.

We first considered that $FANC^{-}$ cells have a higher mutation rate than $FANC^{+}$ cells (39–41). As the quantitative increase in mutation rate is not well-established, we reran the experiments above to determine outcomes if TP53 mutation rates in uncorrected $FANC^{-}$ cells ($\mu_{FANC^{-}}$) were 1.5-, 2-, 4-, or 8-fold higher than in corrected $FANC^{+}$ cells ($\mu_{FANC^{+}}$, Figure 4DG). In the absence of gene correction, when $\mu_{FANC^{-}}$ was increased by a factor of eight, there was a corresponding increase in the tissue burden of ${TP53}^{-}$ cells to cover 23% of tissue sections at 46 years compared to only 2.7% under the background mutation rate (Figure 4D). While elevated mutation rates produced more ${TP53}^{-}$ clones, individual clone sizes remained similar to those under baseline mutation rates (Supplemental Figure 2AB). We found that by introducing gene correction with a small persistence coefficient ($p_{\text{corr}}=0.01$, Figure 4D), ${TP53}^{-}$ tissue coverage was modestly reduced compared to uncorrected tissue (a reduction of 42% in simulations in which $FANC^{-}$ cells had a 8x elevated mutation rate). In contrast, gene corrections with a larger persistence coefficient ($p_{\text{corr}}=0.1$, Figure 4D) resulted in tissue sections with almost no additional TP53 mutations above baseline simulations performed using background mutation rates. This effect depended on gene-corrected $FANC^{+}$ cells replacing more mutable $FANC^{-}$ cells: in the 2% of $p_{\text{corr}}=0.1$ simulations in which gene correction was stochastically lost, $FANC^{-}/{TP53}^{-}$ cells expanded as if they were in uncorrected tissue (see panel inset in Figure 4G). Full 46-year tissue coverage trajectories from these simulations are shown in Supplemental Figure 1D-F.

We next considered that TP53 mutations might confer a stronger proliferative advantage in FA cells than in gene-corrected cells (i.e., $p_{FANC^{+}}^{TP53^{-}}<p_{FANC^{-}}^{TP53^{-}}$). To test the impact of this effect, we reran the experiments above with identical $FANC^{-}$ and $FANC^{+}$ mutation rates, but with $p_{FANC^{-}}^{TP53^{-}}$ increased by a factor of 1.5-, 2-, or 4-fold compared to $p_{FANC^{+}}^{TP53^{-}}$ (Figure 4EH). In the absence of gene correction, when $p_{FANC^{-}}^{TP53^{-}}$ was increased by a factor of four, there was a corresponding increase in the tissue burden of ${TP53}^{-}$ cells to cover 33% of tissue sections at 46 years compared with 2.7% under the background proliferative advantage. In contrast to the mutation rate experiments, increased ${TP53}^{-}$ coverage was primarily driven by larger clones, rather than greater clone numbers, although there was a modest increase in the number of clones as well (Supplemental Figure 2CD). As was the case for gene correction affecting mutation rates, gene correction with a small persistence coefficient ($p_{\text{corr}}=0.01$, Figure 4E) modestly reduced the ${TP53}^{-}$ tissue burden by ~31% among cells with a 4x elevated proliferative advantage compared to uncorrected tissue. Gene correction with a large persistence coefficient ($p_{\text{corr}}=0.1$, Figure 4E) resulted in tissue sections with almost no excess ${TP53}^{-}$ mutational coverage beyond baseline simulations in which $FANC^{-}$ and $FANC^{+}$ cells have identical persistence coefficients associated with TP53 loss. Again, the rapid spread of gene-corrected cells largely abrogated the proliferative advantage of TP53 mutations in a $FANC^{-}$ background. Full 46-year tissue coverage trajectories are shown in Supplemental Figure 1G-I. These results collectively suggest that mucosal gene therapy has the potential to reduce TP53-associated HNSCCs in FA, where its effectiveness will depend on the spread of gene-corrected cells to counteract FA-associated elevated mutation rates and/or selective pressures.

## Discussion

Our computational model provides several important insights into the practicality and implementation of oral mucosal gene correction: 1) gene correction needs to confer a strong proliferative advantage to escape stochastic loss and to promote tissue replacement on clinically-relevant decadal timescales; 2) tissue replacement success can be maximized by increasing delivery dose to avoid stochastic loss of corrected lineages, and by distributing tissue delivery spacing to reduce competitive interference among corrected lineages; and 3) gene therapy will be most effective in reducing HNSCC risk if it lowers the mutation rate and/or proliferative advantage of oncogenic mutations.

Of note, we found that cells with a strong proliferative advantage (i.e., $p_{\text{corr}}\geq 0.1$) are best poised to survive and then spread rapidly within the oral epithelium. Thus the consequences of gene correction must be persistent, substantial and likely early to have a strong likelihood of countering HNSCC disease risk or progression. Strong proliferative advantages of FA gene correction to confer these benefits are plausible, as evidenced by spontaneous revertant patches in other squamous epithelial diseases such as epidermolysis bullosa. For example, individuals with epidermolysis bullosa can have revertant skin patches 2 cm^2^ or larger even at ages as young as 8, and with geometries inconsistent with emergence during development (23–25). Individuals with ‘ichthyosis-with-confetti’ have large revertant skin patches caused by mitotic recombination that can reach up to 4 cm^2^ in size and have been documented in individuals as young as 18 (42). Moreover, human mucosal epithelium has faster measured cell turnover than does epidermis (36,43–45). This faster turnover could permit even a weak cellular advantage to spread on similar timescales. If FA gene correction confers only a weak mucosal fitness advantage, this advantage might be further augmented by engineering additional genetic alterations during transgene delivery. For example, recent studies have found that *NOTCH1* mutations, despite conferring fitness advantages that promote clonal expansion, minimally alter cancer risk and thus could be protective (20).

Our model predicts that gene correction will be most beneficial at suppressing cancer risk if it reduces the production and spread of pathogenic mutations in an FA background. FA cells accumulate chromosomal aberrations (9) due to DNA damage repair deficits (46–48). Gene correction (15–19) and spontaneous genetic reversion (49–55) in FA patient HSCs have been shown to reduce DNA damage sensitivity and genomic instability. We have less direct evidence that pathogenic mutations like those in TP53 spread faster on an FA background, or that FA gene correction can suppress this advantage. We hypothesize that disruptions to TP53 could impact cells with compromised FA pathway function based on interactions between the pathways. In FA cells, persistent DNA damage activates TP53, halting cell proliferation to allow for DNA damage repair (56,57). Loss of TP53 enables cells to bypass proliferation arrest, potentially providing a selective advantage (58,59). Gene therapy that restores FA pathway function and enables effective DNA repair could thereby lessen the selective pressure for TP53 mutations.

In light of these new insights, we note several places where our model could be further improved or extended. First, we could more explicitly model the stem cell hierarchy within the basal layer. While this would improve the biological accuracy of the model, global tissue dynamics are unlikely to be substantially altered (35). Second, we use a relatively simple proliferative advantage model for TP53 mutations acting through a persistence coefficient, as opposed to the repair-mediated survival benefit explored by Schenk et al. (35). We also only examined single TP53 mutations without considering the effect of homozygous TP53 mutations. However, we note that many TP53 mutations can act in dominant fashion (60,61). Moreover, heterozygous TP53 mutants undergo clonal expansion in mice, and loss of the second allele increases genome instability with little change in cellular proliferation (21). Our use of clinical data and realistic estimates of mutation rates and allelic spread lead us to believe our mucosal model recapitulates expected TP53 dynamics in mucosal epithelia. Future modeling efforts could further explore gene correction with more complicated multi-hit phenotypes and associated clonal expansion processes. Third, we considered only the simplest form of microneedle gene delivery without exploring the many modifiable features of this or of competing gene delivery options (26–31). Modeling that leverages gene delivery platform-specific features should further improve transgene delivery, persistence, and spread.

Gene therapy represents a promising intervention to treat a wide range of disease states. As illustrated here, computational modeling provides a useful framework in which to identify important determinants of therapeutic success across many diseases and their target cells, tissues or organs. A critical next step in determining the potential of FA oral epithelial gene therapy specifically is to quantify the proliferative advantage of gene-corrected cells in FA epithelium. Proliferative advantage might be possible to estimate now in some FA patients by using deep sequencing of oral mucosal biopsies to quantify spontaneous revertant mutations. The number and nature of these revertants could then be used to estimate reversion rates and the likely proliferative advantage of revertant (or, by extension, gene-corrected) cells in FA oral mucosa. These data may also allow a quantitative estimate of the fitness advantages of driver mutations like TP53 in FA oral mucosa. Several new mouse models of FA (see, e.g., (62)) should soon allow both experimental and computational predictions to be tested to quantify the fitness effect of FANC reversion mutations or gene correction. Mouse models have the added advantage of facilitating the tracking of gene-corrected cells to assess the spatial distribution and spread of FA-corrected lineages *in situ*. All of these approaches should provide new insight into the competitive dynamics of FA gene-corrected cells, and allow us to develop better protocols to optimize gene delivery and design clinical trial protocols to ensure success with maximum therapeutic benefit, especially when acting in concert with computational models such as the one presented here.

## Materials and methods

### Overview of the model

#### Model Framework:

We extended a cellular automaton model of epithelial cell division and homeostasis in the Hybrid Automata Library (63), HomeostaticEpidermis (HE) (35), to simulate the dynamics of oral epithelial cells and corrective gene therapy. Briefly, HE simulates cells as independent agents on a three dimensional lattice that divide and die according to the available concentration of a growth factor diffusing from the basal layer (see (35) for full parameterization of this growth factor gradient). During a cellular division event, one daughter cell occupies the location of the parent cell, while the other is positioned either vertically above or in one of the four orthogonal neighboring positions within the basal layer Von Neumann neighborhood. The placement of this second daughter cell is governed by the division location probability ω, where ω=0.2518617 represents the probability of vertical placement, and each orthogonal direction has a probability of (1-ω)/4. If a daughter cell occupies a neighboring position within the basal layer, the displaced cell stratifies out of the basal layer and all cells above it are displaced upwards. The epithelial dynamics of this study focus only on the basal layer, as once a cell stratifies to the suprabasal layers, cells do not de-differentiate and re-enter the basal layer. Cells can mutate during division, which we examine in the latter half of our study. This model reproduces key behaviors of epithelial tissues, including the distribution of mutated clone sizes over time. As such, most existing model parameters are kept at their default values to preserve homeostatic tissue function. New functions and parameters specific to our model are described below.

In our model, all cells begin with mutant FANC gene function (i.e., an FA phenotype), as expected in an inherited disorder. Throughout most of the analyses, this FA phenotype does not affect the normal maintenance of homeostatic equilibrium or cellular behavior, although in section *Lineage competition between gene-corrected cells and TP53 mutants*, we describe how uncorrected FA cells may affect genome stability and control of ${TP53}^{-}$ mediated clonal expansions.

### Adaptation of epidermal model for the oral epithelium

We sought to preserve homeostatic behavior of the HE model while accounting for the increased turnover rate of oral epithelial cells compared to epidermal cells. Under the default parameters in the HE model, basal cells divide on average 0.4 times per week (Supplemental Figure 3). However, basal cells in the oral epithelium and esophagus divide approximately twice per week (21,36,43). We therefore rescaled time in our model such that each reported year of simulation time in the oral epithelium corresponds to 4.5 years of simulation time in the HE model.

To reflect the higher density of cells in the oral epithelium versus epidermal tissue, tissue section sizes are determined using a density of 15,000 basal layer cells/mm^2^ (22,36), instead of 10,000 basal layer cells/mm^2^ in the HE model (35,64).

#### Gene Correction:

To mimic *in vivo* microneedle gene delivery that corrects FA oral epithelial cells, we allowed FA cells to be transformed into corrected cells by exposure to a simulated transgene at a pre-specified treatment time. We apply the *in silico* transgene delivery to the center of simulated tissue sections except when otherwise noted at the start of the simulation. Transgene spread through the tissue is governed by an approximation to Brownian diffusion away from the injection site. Specifically, for each cellular position on the tissue basal layer, we determine the relative probability of correction by integrating the PDF of a bivariate Gaussian distribution over the cell’s x and y coordinate boundaries on the lattice. We then sample k cell coordinates for correction from this probability distribution without replacement. To simulate a transgene injection with greater or lesser diffusion, we examine Gaussian distributions with different variances (D) centered on the injection site coordinates ($x_{inj},y_{inj}$) as follows:

$$\mu =\left(\begin{array}{l} x_{inj}\\ y_{inj} \end{array}\right),\Sigma =\left(\begin{array}{ll} D & 0\\ 0 & D \end{array}\right).$$

Corrected cells can have altered dynamics in the basal layer that may allow them to spread, in analog to the ability of gene-corrected cells to spread in the hematopoietic compartment. In stratified squamous epithelium, clonal expansion occurs due to an imbalance in the fate of progenitor cells in the basal layer (22,36,38). We model this mechanism via a persistence coefficient ($p_{\text{corr}}$), which prevents a cell from being displaced by its neighbor cell’s division. Specifically, when a cell divides and randomly chooses to displace a neighboring cell possessing a persistence coefficient $p_{\text{corr}}$, the neighboring cell will not be displaced vertically with probability $p_{\text{corr}}$, and will be displaced vertically with probability $1-p_{\text{corr}}$. If the neighboring cell is not displaced, the dividing cell instead places its daughter cell vertically and does not displace any neighboring cells. This mirrors the implementation of *NOTCH1* mutations in Schenck et al. (35). FA cells are assumed to have $p_{\text{corr}}=0$ throughout.

### Interrogating clonal expansion of corrected cells in an FA background

To investigate the spread of gene correction through the oral epithelium, we simulated FA tissue sections of size 0.67 mm^2^ (100 cells × 100 cells). We introduced a single corrected patch of k cells with persistence coefficient $p_{\text{corr}}$ in the center of the basal layer of the tissue as described above and recorded tissue states longitudinally for up to 50 years at six month intervals. Unless otherwise noted, k=10 and D=2, reflecting a tightly clustered patch of few corrected cells. Simulations were stopped if no corrected cells remained in the basal layer (‘loss’) or if the corrected patch of cells occupied 80% of the basal layer (‘confluence’). Simulations reaching neither endpoint in 50 years were designated as ‘ongoing’. We ran 100 correction replicates for $p_{\text{corr}}=(0,0.001,0.01,0.1,0.2,0.5,1.0)$ and recorded the proportion of simulations in which correction was lost or reached confluence and the time at which that event occurred. We repeated these analyses examining different numbers of corrected cells (k=3,10,30) and different degrees of transgene diffusion (D=2,10,20). Clone size distributions were determined by computing the number of cells associated with each of the corrected patches for each persistence coefficient after one year. To determine the expansion rates of corrected patches, we examined only simulation replicates reaching confluence and converted the number of corrected cells at each timepoint (six-month intervals) into area assuming each cell was 66.7 μm^2^ (22,36).

### Lineage competition between gene-corrected cells and TP53 mutants

We investigated how gene correction might decrease the prevalence of TP53 mutant cells in the oral epithelium of people with FA. To run these analyses, we expanded our model to permit cells to acquire TP53 inactivating mutations at cell division in both ${\text{FANC}}^{-}$ (FA) and $FANC^{+}$ (corrected) cells. Each cell division, each ${\text{FANC}}^{-}$ and $FANC^{+}$ cell acquires an expected number of TP53 inactivating mutations, $\mu_{FANC^{-}}$ and $\mu_{FANC^{+}}$, respectively. We determined the probability of TP53 inactivation to be $7.48*10^{-7}\;{\text{division}}^{-1}$ as the product of the normalized genewide TP53 mutation rate $2.99*10^{-6}\;{\text{division}}^{-1}$ (35,65) and the probability of gene inactivation conditional on mutation as determined from deep mutational scanning data (~0.25, (66)). More information on this calculation is available in ‘Parametrizing the proliferative advantage and mutation rate of *TP53*^−^- mutations’. A Poisson number of inactivating mutations with $\lambda =\mu_{FANC^{-}}$ or $\lambda =\mu_{FANC^{+}}$ is drawn for each cell division and cells gain a fitness advantage if an inactivating mutation occurs. Acquiring multiple mutations in TP53 did not alter clonal fitness beyond a single hit (see Discussion). ${\text{FANC}}^{-}$ and $FANC^{+}$ cells containing a TP53 inactivating mutation had an additional increase in their persistence coefficient of $p_{FANC^{-}}^{TP53^{-}}$ and $p_{FANC^{+}}^{TP53^{-}}$, respectively. Initially, we assumed that $p_{FANC^{-}}^{TP53^{-}}=p_{FANC^{+}}^{TP53^{-}}=0.01$, based on model fitting to the prevalence of TP53 mutations in noncancerous tissue (see section ‘Parametrizing the proliferative advantage and mutation rate of *TP53*^−^ mutations’ for full details).

We considered three experimental conditions in which gene correction could slow the rate at which TP53 mutations accumulated in tissue:

1. If $FANC^{+}$ cells were more difficult to displace than $FANC^{-}$ cells, but were otherwise identical in terms of the TP53 mutation rate and clonal expansion speed. In this case, we examined simulation runs in which $\mu_{FANC^{-}}=\mu_{FANC^{+}}$ and $p_{FANC^{-}}^{TP53^{-}}=p_{FANC^{+}}^{TP53^{-}}$.
2. If $FANC^{+}$ cells were more genetically stable than $FANC^{-}$ cells and thus acquired fewer TP53 mutations. In this case, we examined simulations in which $\mu_{FANC^{-}}=m\cdot \mu_{FANC^{+}}$ and $p_{FANC^{-}}^{TP53^{-}}=p_{FANC^{+}}^{TP53^{-}}$, where m=(1.5,2,4,8).
3. If $FANC^{-}$ cells permitted a faster rate of clonal expansion of TP53 inactivating mutations than $FANC^{+}$ cells. In this case, we examined simulations in which $\mu_{FANC^{-}}=\mu_{FANC^{+}}$ and $p_{FANC^{-}}^{TP53^{-}}=r\cdot p_{FANC^{+}}^{TP53^{-}}$, where r=(1.5,2,4).

We conducted 300 replicates of experimental condition 1 and 100 replicates of experimental conditions 2 and 3, both with and without gene correction. Among simulations with gene correction, we examined small and large persistence coefficients for corrected cells ($p_{\text{corr}}=0.01$ and $p_{\text{corr}}=0.1$, respectively). We ran simulations on 0.33 mm^2^ (70 × 70 cells) tissue sections for up to 50 years with k=30 initially corrected cells distributed with D=2. Simulations without correction ran uninterrupted for the full 50 years. Simulations with correction were run for up to 50 years, but were stopped if all corrected cells were lost from the basal layer. This stoppage criterion reflects our assumption that once all corrected cells are lost from the basal layer, the tissue behaves similarly to one never having undergone correction. To address variation in replicate numbers caused by early stoppage, mutational characteristics for samples in which gene correction was lost were sampled from a random replicate that did not undergo correction at each timepoint. We then quantified the proportion of the tissue with at least one TP53 mutation at six month intervals for up to 46 years.

### Parametrizing the proliferative advantage and mutation rate of ${TP53}^{-}$ mutations

To quantify how gene correction could disrupt TP53 mutation clonal expansions in FA oral epithelium, we first parameterized ${TP53}^{-}$ mutation rates ($\mu_{FANC^{+}}$) and proliferative advantages ($p_{FANC^{+}}^{TP53^{-}}$) in the context of our model. We estimated these parameters via comparison to a published study of TP53 mutational expansion in healthy (i.e., non-FA) esophagus (67).

Martincorena et al. (67) collected 2 mm^2^ sections of esophageal tissue from study participants of different ages and deep sequenced each section to measure the number of unique TP53 mutations and their corresponding variant allele frequencies (VAFs). Because 2 mm^2^ tissue sections are computationally expensive to simulate under the HE model, we simulated smaller tissue sections (70 × 70 cells or ~0.33 mm^2^) and compared them to a spatially downsampled version of (67), intended to mimic results from Martincorena et al. if smaller sections were sequenced.

To approximate downsampling of the clinically-derived 2 mm^2^ samples, we first spatially reconstructed possible tissue sections that reflect the mutation data observed in each full 2 mm^2^ sample (i.e., each tissue section at a given age has i distinct TP53 mutations indexed 1,…,i, at frequencies $f_{1},\ldots ,f_{i}$). With our expected cellular density, each 2 mm^2^ tissue section is represented by a 173 × 173 cellular grid. We then place i mutations in cells on the grid such that each mutation is spatially contiguous and at its observed frequency, $f_{i}$. Specifically, starting with the most frequent mutation, we choose a random starting point on the grid to assign the mutation and then mutate a random neighboring cell of the currently mutated cells to extend the spatially contiguous mutational patch until the mutation reaches its observed frequency $f_{i}$. This process is repeated serially for each additional mutation observed in a tissue section, with the additional constraint that if the frequency of total mutations reaches 0.5 or more, the mutation that would exceed this threshold is instead assigned to an existing mutant clone background of higher frequency. After reconstructing potential tissue sections that could produce the correct number of TP53 mutations and their VAFs in the 2 mm^2^ sections, we randomly sampled a 0.33 mm^2^ (70 × 70 cell) square subsection from each larger reconstructed sample and counted the number of mutations and their VAFs in this subsection. To align with Martincorena et al. (67), who excluded mutations sampled at frequencies below 0.0018 (108 genomic copies/(29929 cells * 2 genomic copies/cell)) in 2 mm^2^ tissue sections, we applied the equivalent threshold in our 0.33 mm^2^ sections, corresponding to a frequency 0.011 (i.e., 108 genomic copies/(4900 cells * 2 genomic copies/cell)).

To identify the TP53 persistence coefficient value that most accurately reflects the mutation patterns observed in empirical data, we simulated 70×70 tissue sections across a range of $p_{FANC^{+}}^{TP53^{-}}$ values and compared the simulated data with the downsampled empirical observations. Martincorena et al. (67) collected 844 samples from nine donors of varying ages. We examined the 560 samples from the six donors aged 20 to 55 to match the correction timescales profiled in this study. 177 samples contained detectable TP53 mutations, which were spatially reconstructed and then downsampled as described above. Only TP53 nonsense, missense and synonymous SNV mutations were included, with non-SNV and splice mutations excluded. Total empirical mutational prevalences (i.e., the number of unique TP53 mutations per tissue) were computed by combining the downsampled tissue sections and the tissue sections containing no TP53 mutations.

We conducted 100 simulation replicates for each $p_{FANC^{+}}^{TP53^{-}}=(0,0.007,0.01,0.0125,0.015)$ and matched two metrics between the simulated and observed data: the number of unique TP53 mutations in the tissue and the average mutational VAF of an observed TP53 mutation. Using the default mutation rate of the HE model resulted in an excess number of TP53 mutations compared to empirical data, even when these mutations conferred no proliferative advantage (Supplemental Figure 4A). To better align the number of mutations in a tissue sample with observed data, we reasoned that the mutation rate producing mutations capable of expanding was likely lower than expected from the gene length and average mutation rate alone. To compute an adjusted mutation rate, we referenced deep mutational scanning data of TP53 and found that approximately 25% of TP53 mutations were expected to confer a selective benefit (66). We therefore reduced the model’s default expected number of TP53 mutations by a factor of four, and found that this reduced mutation rate ($7.488^{*}10^{-7}\;{\text{division}}^{-1}$) was capable of producing mutation counts and sizes that roughly matched Martincorena et al. (67), as shown in Supplemental Figure 4B.

To compare the number of distinct mutations arising, we calculated the squared differences between the mean number of mutations in the downsampled empirical data and the mean number of mutations in the simulated data for each timepoint and for each $p_{FANC^{+}}^{TP53^{-}}$ value. We then averaged these squared differences across all timepoints and identified which $p_{FANC^{+}}^{TP53^{-}}$ value minimized the mean squared error (Supplemental Figure 4D).

To find the $p_{FANC^{+}}^{TP53^{-}}$ value best able to produce empirical TP53 mutational size distributions (i.e. VAFs), we took a two step approach. First, we fit exponential distributions that described the simulated VAFs for each $p_{FANC^{+}}^{TP53^{-}}$ value separately at each timepoint. We fit these distributions using the R fitdist function within the fitdistrplus package (68). Before fitting, we recentered the simulated VAFs by subtracting off the lower limit of detection frequency (0.011) to better conform to the shape of the exponential distribution (i.e., bounded below at 0). Second, we then used the best fit λ at each time point and for each simulated $p_{FANC^{+}}^{TP53^{-}}$ to compute the probability that an interval encompassing each empirical downsampled VAF measurement was drawn from each of the best fit distributions. Specifically, for an observed VAF measurement f and best fit parameter λ for a given age group and $p_{FANC^{+}}^{TP53^{-}}$, we computed the probability of the observed data as:

$$p\left(f|\lambda \right)=\int_{f-\varepsilon }^{f+\varepsilon }\lambda e^{-\lambda x}dx.$$

We used ε=0.0002 although we found that results were robust to our choice of small ε. As when fitting the distributions initially to find the best fit λs, we recentered the observed VAFs by subtracting off the lower limit of detection before evaluating the probability expressions above. We then summed the log probabilities for a given $p_{FANC^{+}}^{TP53^{-}}$ across all age groups to find the $p_{FANC^{+}}^{TP53^{-}}$ that generates simulated data that best conforms to the empirical data through time (Supplemental Table 1, Supplemental Figure 4CE). We did not perform exponential best fit calculations to $p_{FANC^{+}}^{TP53^{-}}=0$ as there was only one observed mutation for the age ranges 20–23 and 24–27. Two outlier measurements diverging substantially from the overall temporal signal were excluded when determining the best fit parameters: the number of mutations among ages 44–47 and the variant allele frequencies among ages 20–23 (Supplemental Figure 4BC).

We found that $p_{FANC^{+}}^{TP53^{-}}=0.01$ both minimized the squared error for the number of mutations and minimized the negative log-likelihood fit to the mutational VAFs, so we proceeded with $p_{FANC^{+}}^{TP53^{-}}=0.01$ as our proliferative advantage of TP53 mutations in healthy tissue.
